# Microbiological Biosafety, Multiple Drug Resistance and Functional Diversity of Bacteria Associated with the Surfaces of Raw Vegetables

**Published:** 2017-03

**Authors:** Farooq NISMA, Raheem ASIF, Ali BASHARAT

**Affiliations:** Dept. of Microbiology and Molecular Genetics, University of the Punjab, Lahore, Pakistan

## Dear Editor-in-Chief

Fresh agricultural produce can harbor pathogenic microorganisms that can cause serious illnesses when transmitted to the human beings after consumption. The frequency of outbreaks from the consumption of contaminated fresh vegetables and fruits has amplified in recent decades (1). True and opportunistic human pathogens have been reported with foodborne outbreaks due to their continuously increasing adaptation to soil and plant associated environments. They may be highly competitive for nutrients and use their ability to produce antimicrobial metabolites to suppress the native microflora and excel in colonization and proliferation on plant surfaces. For example, pathogenic strains of *Pseudomonas aeruginosa* and *Stenotrophomonas* are well documented to colonize plant surfaces (2). Equal ability of *Burkholderia cepacia* to cause infections in plants and humans has been reported (3).

Therefore, the main objective of this study was to evaluate the microbiological biosafety of the raw-eaten fresh vegetables by exploring phylogenetic and functional bacterial diversity.

Thirty-six samples of raw-eaten fresh vegetables including carrot, cabbage, and turnip (12 samples each) were collected from 12 different sites in Lahore, Pakistan in 2015. Strains of gram-positive and gram-negative bacteria isolated from different vegetable samples are listed in Table 1 and 2.

**Table 1:** 16S rRNA gene sequencing of Gram-positive bacteria isolated from different vegetables

**S. No.**	**Isolates**	**Identified as**	**Accessions**
1	BPc-4	*Bacillus cereus* BPc-4	KJ865556
2	BPt-5	*Staphylococcus equorum* BPt-5	KJ865579
3	BPb-5	*S. aureus* BPb-5	KJ865591
4	EMc-3	*B. anthracis* EMc-3	KJ865553
5	Eb-9	*B. cereus* Eb-9	KJ865594
6	Eb-10	*B. thuringiensis* Eb-10	KJ865560
7	Lt-41	*S. xylosus* Lt-41	KJ865585
8	Lt-73	*S. warneri* Lt-73	KJ865565
9	Lb-41	*Arthrobacter nicotianae* Lb-41	KJ865583
10	Lb-61	*B. subtilis* Lb-61	KJ865595
11	LCw-22	*B. cereus* LCw-22	KJ865598
12	MCb-3	*S. arlettae* MCb-3	KJ865592
13	MCb-4	*Exiguobacterium mexicanum* MCb-4	KJ865577
14	MCb-6	*B. cereus* MCb-6	KJ865559
15	MCb-8	*B. subtilis* MCb-8	KJ865584
16	MSt-1	*S. xylosus* MSt-1	KJ865600
17	MSt-3	*S. gallinarum* MSt-3	KJ865580
18	MSt-7	*B. cereus* MSt-7	KJ865557
19	MSt-8	*B. cereus* MSt-8	KJ865589
20	MSb-3	*B. cereus* MSb-3	KJ865596
21	MSb-4	*B. anthracis* MSb-4	KJ865558
22	MSc-5	*S. warneri* MSc-5	KJ865590
23	PCt-1	*B. cereus* PCt-1	KJ865573
24	Xt-1	*S. xylosus* Xt-1	KJ865597
25	Xt-6	*Lysinibacillus fusiformis* Xt-6	KJ865555
26	Xb-6	*B. anthracis* Xb-6	KJ865582
27	Xc-7	*L. fusiformis* Xc-7	KJ865599

**Table 2:** 16S rRNA gene sequencing of Gram-negative bacteria isolated from different vegetables

**S. No.**	**Isolates**	**Identified as**	**Accessions**
1	BPc-1	*Serratia rubidaea* BPc-1	KJ865576
2	BPc-3	*Pantoea dispersa* BPc-3	KJ865552
3	BPb-3	*Acinetobacter calcoaceticus* BPb-3	KJ865562
4	Eb-1	*Klebsiella penumoniae* Eb-1	KJ865601
5	Eb-2	*P. vagans* Eb-2	KJ865561
6	Eb-4	*A. calcoaceticus* Eb-4	KJ865586
7	Eb-6	*Burkholderia cepacia* Eb-6	KJ865578
8	Eb-8	*A. calcoaceticus* Eb-8	KJ865568
9	EMt-1	*A. bouvetii* EMt-1	KJ865593
10	EMt-5	*Enterobacter amnigenus* EMt-5	KJ865563
11	EMc-2	*S. rubidaea* EMc-2	KJ865581
12	EMc-4	*A. calcoaceticus* EMc-4	KJ865567
13	Lc-52	*A. calcoaceticus* Lc-52	KJ865566
14	Lcr-22	*S. rubidaea* Lcr-22	KJ865575
15	MCt-1	*Stenotrophomonas maltophilia* MCt-1	KJ865603
16	MCt-5	*Kluyvera cryocrescens* MCt-5	KJ865554
17	MCt-6	*S. ureilytica* MCt-6	KJ865570
18	Mc-2	*S. rubidaea* Mc-2	KJ865602
19	Mc-3	*St. maltophilia* Mc-3	KJ865587
20	Mc-4	*A. calcoaceticus* Mc-4	KJ865588
21	MSt-6	*A. calcoaceticus* MSt-6	KJ865569
22	MSc-1	*Pantoea* sp. MSc-1	KJ865571
23	PCt-2	*E. cloacae* PCt-2	KJ865574
24	Xc-3	*E. cloacae* Xc-3	KJ865572
25	Xc-5	*Pseudomonas putida* Xc-5	KJ865551
26	Xc-6	*Citrobacter freundii* Xc-6	KJ865549
27	Xt-3	*C. werkmannii* Xt-3	KJ865550
28	Xb-3	*S. rubidaea* Xb-3	KJ865564

Bacterial genera *Bacillus, Staphylococcus, Acinetobacter,* and *Serratia* were commonly associated with all the three vegetables (Carrot, turnip, and cabbage). Other bacterial groups including *Lysinibacillus, Stenotrophomonas, Citrobacter* and *Enterobacter* were commonly isolated from carrot and turnip samples. Some bacterial genera were found specifically associated with cabbage (*Exiguobacterium, Arthrobacter, Burkholderia,* and *Klebsiella*), turnip (*Kluyvera*) and carrot (*Pseudomonas* and *Pantoea*). Although no obligate human pathogens were detected in our vegetable samples but some potentially pathogenic bacteria were isolated that included *B. cereus, B. anthracis, S. aurues, E. cloacae, E. amnigenus* and *K. Pneumoniae* (Table 1, 2)*.*

Gram-positive bacteria showed high resistances of 52% (amoxicillin) and 59% (nalidixic acid) against two broad spectrum antibiotics. Bacterial strains associated with fresh agricultural produce also exhibited beneficial plant growth promoting attributes; especially, IAA production and biofilm formation. A variety of (IAA) producing bacterial strains has been shown to harbor by plants that positively influence plant growth and productivity (4). Biofilm formation on plant surfaces may be associated with symbiotic or pathogenic response depending on microbial species (5).

Fresh vegetables from different localities of Lahore were inhabited by potential human pathogens that make the biosafety of this vegetable questionable. Nevertheless, due to close proximity with the plant surfaces, these microbes also harbor beneficial plant growth promoting traits; especially auxin production.
